# The Effect of Sociodemographic Factors on Obtaining a Place in Core Surgical Training

**DOI:** 10.7759/cureus.31271

**Published:** 2022-11-08

**Authors:** Marwan Sarsam, Martina Spazzapan, Lalana Songra, Zaina Salahuddin, Gargi Pandey, Bryan Chew, Hayley Magill

**Affiliations:** 1 Trauma and Orthopaedics, University College Hospital, London, GBR; 2 Urology, Princess Royal University Hospital, London, GBR; 3 Urology, Whittington Hospital, London, GBR; 4 Trauma and Orthopaedics, Kings College Hospital Trust, London, GBR; 5 Otolaryngology, Barts Health NHS Trust, London, GBR; 6 Trauma and Orthopaedics, Royal National Orthopaedic Hospital, London, GBR; 7 Plastic Surgery, St George's Hospital, London, GBR

**Keywords:** surgery application, surgery interviews, surgery, core surgical training, education

## Abstract

Competition for Core Surgical Training (CST) is rising, placing a strong emphasis on interview performance. Several interview courses offer to help candidates secure their chosen surgical job but at premium fees. A group of London-based CSTs started a free course offering high-quality mock interview experiences to over 90 applicants in 2022, with the aim of providing an accessible opportunity for financially disadvantaged candidates.

Course candidates completed three sets of questionnaires, pre- and post-mock interview, and a final one upon job allocation. Candidates’ educational background and schooling history were obtained as well as their self-assessment score, eventual rank after interview and the rank of the job they had accepted.

The three sets of questionnaires were completed by 87, 73 and 45 candidates respectively. Overall, there was a statistically significant difference in self-reported confidence scores after the course (P < 0.001). There was no significant difference in the self-assessment score of the 44.2% of candidates who had attended private education in the UK, compared to publicly educated (P = 0.0525), nor was there a difference in their rank after interviews (P = 0.236). Candidates who spent £50 or more had higher self-assessment scores (P = 0.042) but they didn’t rank higher in overall scores (P = 0.591).

Interview preparation courses are helpful in increasing candidates’ confidence, however spending more money does not translate into a better overall interview performance. Our study suggests that candidates from private education backgrounds do not have an advantage in the CST application process.

## Introduction

Surgical training has been historically recognised as a competitive programme, attracting highly motivated and driven individuals. Upon graduation, medical doctors in the United Kingdom (UK) complete two years of Foundation Training to gain generic competencies. They then undergo national selection to obtain a place on a two-year Core Surgical Training (CST) programme, followed by a further round of national selection for Higher Surgical Training (HST) in their preferred specialty.

Entry into CST is competitive, with an applicant-to-offer ratio of 4.16 in 2021, and approximately 600 trainees entering CST in the UK annually [1]. As numbers of applicants increase and the number of available posts decrease, there is an interest in better understanding the applicant population [2].

CST applicants are ranked based on a combined interview and portfolio performance comprising domains such as performance at Royal Colleges examinations, attendance at surgical courses and conferences, and involvement in audit and research. To score highly in these domains and prepare well for interviews, applicants may spend large sums of money. The costs of entry into surgical training have been found to be the highest when compared to other specialties, with the average cost of reaching the desired criteria for specialty training in surgery at £5564, compared to £3869 for medical specialties [3]. 

While entry into surgical training remains competitive, concerns exist regarding barriers to recruiting a diverse surgical workforce. Deterring factors have been identified with regard to surgical training, including a poor work-life balance, financial requirements, and the perception that surgery is a competitive and aggressive career [4]. Addressing both the monetary and non-monetary deterrents of surgical application could potentially make surgical training more accessible. Making surgery more accessible has the potential to nurture advancement in the field [5]. A diverse surgical team will also attract a wider variety of highly competent individuals into the field.

Given the many barriers to accessing CST, the authors of this paper (current core trainees in London) created the Surgical Training Accessible Resources Society (STARS). The aim was to create free resources for CST applicants and study the relationship between sociodemographic factors, expenditure, and outcomes in surgical applications.

Data from this paper is due to be presented as a poster at the ASIT Future Surgery Conference on 15th-16th November 2022.

## Materials and methods

A core surgical interview preparation course was designed by the STARS committee, a team of seven Core Surgical Trainees based in London. A free, initial introductory lecture was delivered virtually in December 2021, available to an unlimited number of applicants worldwide. The lecture was advertised on social media platforms.

Attendees of the lecture were invited to sign up for one of 108 mock interview slots, allocated on a first come first served basis, with preference given to those applying to CST in 2022. Mock interviews were delivered at no cost to interviewees by volunteer core surgical trainees based in London between 15th of December 2021 and 26th of January 2022. Interview groups comprised two interviewers and four interviewees. Each interview lasted 20 minutes, with 10 minutes feedback post-interview. Interviewees were invited to watch the interviews of other candidates in their group, to increase exposure to different scenarios. 

A pre- and post-mock interview questionnaire was sent to candidates for completion. A third questionnaire was sent for candidates to complete following job allocations. All Information collected was anonymised.

Within the pre-mock questionnaire, data were collected on how prepared participants felt for each component of the CST interview. Data was also collected on self-assessment scores; whether they had applied to CST previously, if they had attended other interview courses and, if so, how much money have they spent in total on interview courses. Information regarding type of school attended was also collected. This was split into UK and overseas education. UK education was further split into state school (government funded school), grammar school (government funded but with local entry criteria prioritising the more intellectual children) and private school (education fully funded by child/parents).

The post-mock questionnaire collected feedback on the mock interview process, specifically data on confidence scores post-mock interview, the usefulness of the course and whether they would recommend the course to others, with an option to provide feedback on areas for improvement. Confidence scores were rated from 0 to 5, with 0 representing ‘not prepared at all’ and 5 representing ‘very well prepared’.

The third questionnaire, sent following job allocations, collected data on whether candidates were invited for an interview and, if so, whether they matched with a job on the initial or subsequent allocation rounds. Further data was also collected on the candidates’ interview rank, the rank of the job they matched with and whether they accepted or rejected the offer. Candidates again rated the quality of the mock interviews on a scale of 0 to 5 with regard to how effective they felt it was in getting them their chosen job. 

Statistical analyses were performed regarding the success of the course with confidence scores pre- and post-interview analysed using a 2-tailed t-test with equal variance assumption. A p-value of < 0.05 denotes significance. An independent t-test was also performed to compare course participant portfolio scores and overall ranking, with the amount spent on interview courses (</= £50 or > £51), interviewee schooling history and gender. Fishers exact test was performed to analyse the link between an offer of a core surgical training post and applicant schooling history (UK private vs non-private), the amount spent on interview preparation courses (</= £50 or > £51) and their gender. The relationship between applicant rank and job preference was analysed using logarithmic regression.

No ethical concerns were identified, the Health Research Authority (HRA) online tool was completed and confirmed ethical exemptions for this study. 

## Results

A total of 87 pre-mock interview, 73 post-mock interview and 45 post-job allocation questionnaires were completed. Self-confidence scores post-mock interview were significantly higher across all domains compared to pre-mock interview confidence scores (Table 1). The average overall confidence score pre-mock interview was 2.6 (+/- 0.706) and increased to 3.81 (+/- 0.544) after delivery of the mock interview sessions (p = < 0.001). Scores for overall understanding of the format of the interview increased from 3.52 (+/- 0.975) to 4.68 (+/- 0.497) (p = < 0.001).

**Table 1:** The comparison of pre-mock interview confidence scores vs post-mock interview confidence scores in the various domains of the interview.

Domain	Pre-interview confidence scores	Post-interview confidence scores	P values
Overall	2.6 (+/- 0.706)	3.81 (+/- 0.544)	p = <0.001
Clinical station	2.87 (+/- 0.818)	3.88 (+/- 0.551)	p = <0.001
Management station	2.68 (+/- 0.755)	3.82 (+/- 0.609)	p = <0.001
Leadership presentation	2.27 (+/- 0.773)	3.41 (+/- 0.863)	p = <0.001
Understanding of interview format	3.52 (+/- 0.975)	4.68 (+/- 0.497)	p = <0.001

Seventy-two of 73 (98.6%) of the course participants said that they would recommend the course to others, with one candidate stating they may recommend the course to others. 

Of the interview course participants, 38/86 (44.2%) reported attending a UK private high school. There was no significant difference in self-reported portfolio scores and type of schooling. Of the 77 course participants who provided information on their portfolio scores and schooling history, 35 (45.5%) attended a UK private school and 42 (54.5%) attended a non-private school (state = 24 (31.2%), grammar = 14 (18.2%), overseas = four (5.19%)). The average portfolio score (maximum score is 72 points) for those who attended private school in the UK was 54.4 (+/- 8.09) and 53.3 (+/- 6.55) for those who attended a state, grammar or overseas school (p = 0.525).

Thirty of 87 (34.5%) of the course participants reported spending more than £50 on preparatory core surgical interview courses, with 56/87 (64.4%) spending £50 or less. Seventy-eight course participants submitted responses regarding their spending and portfolio score. Participants who spent £50 or less had significantly lower portfolio score than those who spent more than £50 on interview courses, with those who spent £50 or less reporting an average score of 52.6 (+/- 6.90) and those who spent more than £50 reporting an average score of 56.1 (+/- 7.38) (p = 0.042). Average portfolio score overall was 53.8. 

Overall, of the 86 respondents whose gender was known, 46/86 (53.5%) were male and 40/86 (46.5%) were female. There were 75 cases in which both gender and portfolio score were known, concluding no significant difference seen between males and females for portfolio scores. The average portfolio score of the 34 female respondents was 52.6 (+/- 7.45) and 55 (+/- 6.83) for the 41 male respondents (+/- 7.02) (p = 0.209). 

**Figure 1:**
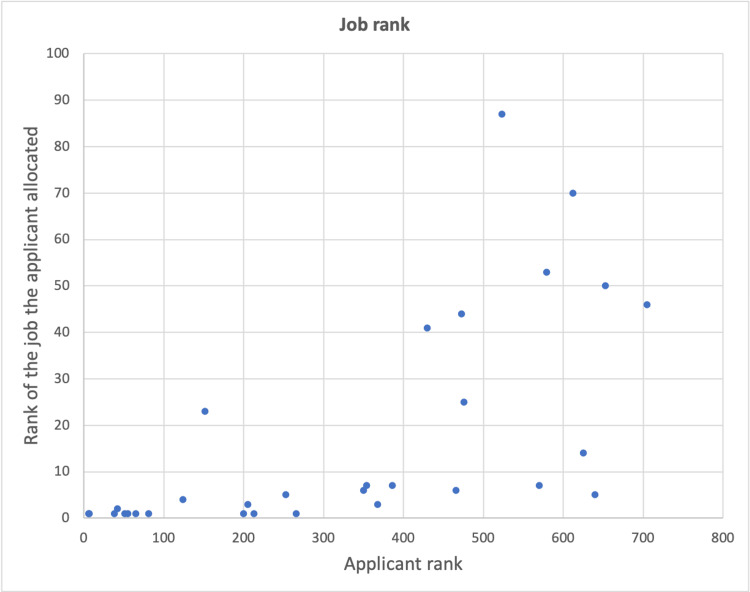
Correlation between overall rank of a candidate and the rank of the job they are allocated on their preferences list. Those who were offered a job they ranked more than 100th were considered an anomaly and excluded.

Forty-five responses were recorded regarding job ranking and receipt of a CST job offer, of which 39 had previously responded with their schooling and spending history. Forty-one of 45 (91.1%) course participants who responded were successful in receiving an offer of a Core Surgical Training post. The average rank of course participants was 418, with 8/45 (17.8%) ranking among the top 100 candidates. The lowest ranking candidate still receiving a job offer ranked 709th. Eleven candidates ranked in the top 200 and all 11 matched with one of their top five jobs (eight matched with their first-ranked job). All 19 candidates who ranked in the top 400 were matched with a job they ranked in their top 10, while those ranking over 400 after interviews had a wider spread, some securing as high as their seventh-ranked job while others only securing their 151st (Figure 1).

There was no significant difference (p = 0.342) in receipt of an offer for a Core Surgical Training post in participants from UK private (16/19), and non-private (19/20) educational backgrounds, nor the average overall ranking of applicants (465 (+/- 262) vs 400 (+/- 294), p = 0.236). No significant difference was seen between participants who spent more or less than £50 on interview preparation courses and overall ranking (465 (+/- 309) vs 414 (+/- 262), p = 0.591) nor was there a difference in likelihood of obtaining a CST post (11/14 vs 24/25, p = 0.123). There was also no significant difference seen between males or females with regards to attainment of a Core Surgical Training post (20/23 vs 18/19 p=0.613), however females on average ranked higher than their male counterparts (345 (+/- 237) vs 495 (+/- 285, p = 0.074). 

Thirty-five of 44 (79.5%) of the interview course participants felt the STARS course helped them get a job or increased their rank to get a better job. 

## Discussion

The STARS preparation course received insightful feedback allowing a thorough evaluation of the free virtual course. It also provided important data to better understand the current cohort of CST applicants. We found that on average, those ranking among the top 200 applicants were able to get one of their top five choices of jobs; those ranking in the top 400 obtained jobs in their top 10 choices. Moreover, results from our study suggest that, while differences in educational background exist among applicants, these do not appear to be associated with differences in attainment of a CST post. 

Educational background and performance of CST applicants 

“Differential attainment at postgraduate assessment is the lens through which we see the accumulation of educational privilege and disadvantage.” Ellis et al. suggests that early educational advantages of private schooling continue to have an impact and are enhanced in post graduate education [6].

Among other indicators, type of schooling has been used as a proxy to infer socioeconomic background in medical education. Private education comprises about 7% of pupils aged five to 18 across the UK. In contrast, roughly a quarter of first year-medical students have had private school education [7]. Kumewnda et al. found that doctors who attended fee-paying schools were significantly more likely to enter surgical training compared to those who attended state-funded schools [8]. Doctors who enter surgical training posts have been shown to be from more affluent socioeconomic backgrounds [9]. This could be attributed to the high costs of surgical training compared to other specialties [3]. In contrast we found no significant difference in portfolio scores, the receipt of a core surgical training post, or average ranking, and type of schooling. It should be considered that high socioeconomic status does not always mandate private school education, especially in the UK, where private schools are not always geographically convenient. 

Gender and performance of CST applicants 

Females currently make up 54% of foundation doctors (doctors in their first two years of medical practice after completing medical school training in the UK), however, they make up only 41% of core surgical trainees, 30% of higher specialty trainees and 12% of consultants [10]. Our results provide an important insight into the gender divide and performance at CST recruitment. In our sample 53.5% of the respondents were male and 46.5% were female. This is in-line with the published demographics showing a lower number of applicants among females. 

No significant differences between gender and portfolio scores were identified. Although females on average had ranked higher than males, we did not find any significant difference with regards to obtaining a CST post. This is in line with existing literature, as females have been shown to have higher application scores and to be significantly more likely than male applicants to be allocated to a higher-choice foundation school [8]. On the other hand, Ellis et al. found that once sociodemographic factors were accounted for, males were significantly more likely to pass MRCS A than females, however not necessarily more likely to pass part B [6]. These results show that while there are gender differences in performance in intercollegiate examinations, overall female participation in surgery remains low. 

Spending, available resources and CST performance

Our results suggest that most CST applicants (65.5%) spent £50 or less on preparatory courses for CST interviews. However, those spending more than £50 on courses had a significantly higher self-assessment score compared to candidates who spent £50 or less. Nevertheless, we found no difference in overall ranking or likelihood of obtaining a CST post in those spending higher sums. STARS course took place prior to candidates being invited to interview; therefore, one suggestion is that those with lower scoring portfolios were less inclined to spend money on interview courses before having a confirmed interview offer. 

Costs of surgical training continue to accrue post completion of CST. A cross-sectional study of surgical trainees in the UK and Ireland estimated that trainees spend between £20,000 and £26,000 in most surgical specialties before completing their training [11]. O’Callaghan et al. showed that surgical trainees spend on average £9105 on courses, £5411 on conferences and £4185 on exams by the end of their training [11]. Only 41% of trainees mentioned that payment for these mandatory training activities was covered at least in part by study budget, raising questions about barriers into recruitment. Awareness of the costs associated with surgical training appears to be limited. A survey of medical students, foundation doctors and core surgical trainees reported more than half of potential applicants surveyed were not aware of the high cost of training [12].

Vinnicombe et al. found that there was an association between familial income and reduced likelihood of individuals in considering a surgical career due to financial cost [12]. The study also showed that respondents who reported receipt of a student loan during medical school were significantly less likely to consider a surgical career. Given the already high costs of surgical training, we provided the course for free to allow accessibility to prospective applicants. This contrasts with some courses which charge up to £397 (Table 2). 

**Table 2:** Available Core Surgical Training (CST) interview courses as of June 2022.

Course Name	Price	Format	Extras
Core Surgery Interview Course (coresurgeryinterview.com)	£397	Online	CV analysis
CST Interview (cstinterview.co.uk)	£150	Online	
CST Secrets (highyielduk.com)	£147	Online	Mock interview demonstration
Interview Skills for Core Surgical Training (RCSEd.ac.uk)	£232	In person	Video with one-to-one feedback
CT/ST Interview Course (medical-interviews.co.uk)	£295	Online	

While delivering the course virtually allowed applicants worldwide to benefit from a free course, it appeared that given there was no financial disincentive for no-shows, attendees might have placed less value in the course as suggested by the number of last-minute drop-outs. In future iterations of the course, we will include a refundable deposit to minimise the impact of last-minute cancellations. However, there are important benefits to running courses virtually, including ease of access and lowered costs for organisers and participants [13].

Limitations

Our study has a number of limitations. Our population of applicants were recruited from social media and word of mouth and therefore may not be representative of the wider cohort of applicants for CST. Further, we had allocated 108 spaces for interviews but only obtained 87 responses for the pre-interview feedback and 72 post-interview ones. Although we collected data on objective measures (such as ranking or offer of a job), other items such as perceived self-confidence were subjective accounts of candidates. Furthermore, it is well known that questionnaires are susceptible to social-desirability bias where participants may feel the need to answer survey questions in a more desirable/positive manner [14]. The authors mitigated this bias by ensuring that the replies were kept anonymous and keeping the questions as neutral as possible. 

## Conclusions

Our pilot year of running a free core surgical training interview preparatory course was successful in not only showing a significant improvement in confidence scores across all domains, but also shedding some light on the current demographics of core surgical training applicants. We found that a high proportion of surgical applicants come from a private education background, however that does not necessarily translate into a surgical training post. This study also highlighted the high financial costs of entering surgical training, which other papers have shown continues throughout training. Given the above, there is a strong argument towards provision of more free courses and resources such as ours to allow core surgical training to be more accessible to everyone.

## Figures and Tables

**Figure 2:**
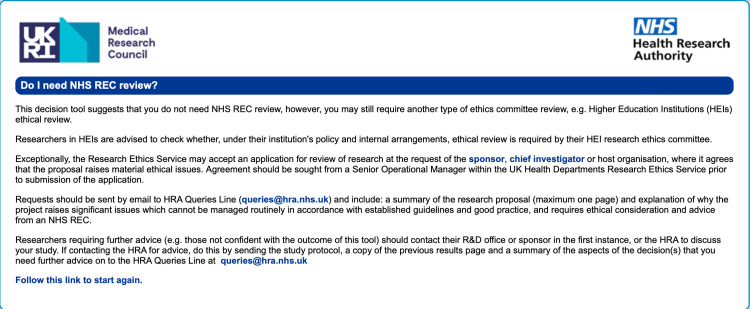
HRA online tool for medical ethics in the UK Confirmation that ethical approval is not required for this paper
